# Genetic Interactions between Brn3 Transcription Factors in Retinal Ganglion Cell Type Specification

**DOI:** 10.1371/journal.pone.0076347

**Published:** 2013-10-08

**Authors:** Melody Shi, Sumit R. Kumar, Oluwaseyi Motajo, Friedrich Kretschmer, Xiuqian Mu, Tudor C. Badea

**Affiliations:** 1 National Eye Institute, National Institutes of Health, Bethesda, Maryland, United States of America; 2 Department of Ophthalmology/Ross Eye Institute, Developmental Genomics Group, and Center of Excellence in Bioinformatics and Life Sciences, University at Buffalo, Buffalo, New York, United States of America; 3 SUNY Eye Institute, Buffalo, New York, United States of America; 4 CCSG Genetics Program, Roswell Park Cancer Institute, Buffalo, New York, United States of America; Virginia Tech Carilion Research Institute, United States of America

## Abstract

**Background:**

Visual information is conveyed from the retina to the brain via 15–20 Retinal Ganglion Cell (RGC) types. The developmental mechanisms by which RGC types acquire their distinct molecular, morphological, physiological and circuit properties are essentially unknown, but may involve combinatorial transcriptional regulation. Brn3 transcription factors are expressed in RGCs from early developmental stages, and are restricted in adults to distinct, partially overlapping populations of RGC types. Previously, we described cell autonomous effects of Brn3b (Pou4f2) and Brn3a (Pou4f1) on RGC axon and dendrites development.

**Methods and Findings:**

We now have investigated genetic interactions between Brn3 transcription factors with respect to RGC development, by crossing conventional knock-out alleles of each Brn3 gene with conditional knock-in reporter alleles of a second Brn3 gene, and analyzing the effects of single or double Brn3 knockouts on RGC survival and morphology. We find that Brn3b loss results in axon defects and dendritic arbor area and lamination defects in Brn3a positive RGCs, and selectively affects survival and morphology of specific Brn3c (Pou4f3) positive RGC types. Brn3a and Brn3b interact synergistically to control RGC numbers. Melanopsin positive ipRGCs are resistant to combined Brn3 loss but are under the transcriptional control of Isl1, expanding the combinatorial code of RGC specification.

**Conclusions:**

Taken together these results complete our knowledge on the mechanisms of transcriptional control of RGC type specification. They demonstrate that Brn3b is required for the correct development of more RGC cell types than suggested by its expression pattern in the adult, but that several cell types, including some Brn3a, Brn3c or Melanopsin positive RGCs are Brn3b independent.

## Introduction

To understand how neuronal circuits function we need to understand the morphological, physiological, molecular and functional properties of the neurons they are made of. To simplify this task, neurons are classified into cell types, under the assumption that each neuronal cell type will have a unique combination of these properties. A good example are Retinal Ganglion Cell (RGC) types, which convey features of visual information (luminosity, color, contrast, motion, etc.) via around 20 distinct parallel channels, to specific retinorecipient nuclei in the brain [1]–[6]. The exact number of RGC types, their properties and the developmental events leading to their specification, are active areas of investigation.

RGC types definitions become far more powerful when physiological, morphological and molecular criteria are combined, and this is greatly facilitated by genetic labeling strategies in mice. Over the last decade, fairly complete characterizations have been achieved for several types of intrinsically photosensitive RGCs (ipRGCs), which can detect light through their own specific opsin, Melanopsin (Opn4), and participate in the circuits for Circadian Photoentrainment, and Pupillary Light Reflex [7]–[12]. ipRGCs [10], [13] do not express either Brn3a or Brn3c [8], [14], and a subpopulation of the M1 ipRGCs which projects to the Suprachiasmatic Nucleus (SCN), is also Brn3b negative and survives deletion of the *Brn3b* gene or ablation of Brn3b positive RGCs [8], [15]. In addition, genetic lines have helped correlate dendritic morphologies, physiological light responses, axonal projections to distinct brain nuclei and in some cases circuit functions for motion sensitive RGCs [15]–[21].

As RGC cell type definitions based on anatomy, physiology and circuit function increase in resolution and coherence, so do our questions regarding the developmental mechanisms generating their diversity. It is generally believed that determination of RGCs as class are under the transcriptional control of the neurogenic bHLH transcription factor Math5 [22], [23], and the POU IV domain transcription factor Brn3b. According to this model, Math5, expressed in retinal precursors and transiently in the postmitotic neurons fated to become RGCs, is controlling the expression of Brn3b, which then in turn activates Brn3a, Brn3c, Eomesodermin [24], Ebfs [25] and maybe other transcription factors to regulate RGC differentiation. However, more recent findings suggest that neither Math5, nor Brn3b are responsible for the entire set of RGC types. Thus, RGCs with well-preserved morphologies can be generated in the absence of either transcription factor [26], and a few RGCs survive combined ablation of both [27]. In addition, Isl1 is required for RGC differentiation in a pathway that is overlapping with Brn3b [28], [29] in a manner analogous to previously noted genetic interactions for the C. elegans and Drosophila homologues of Isl1 and Brn3s. In *C. elegans*, mec-3 (lim homeodomain) and unc-86 (Pou domain) transcription factors function as terminal selector genes regulating touch receptor specific gene programs [30], [31]. In Drosophila, olfactory projection neuron specification/targeting is under the control of a transcriptional code including Islet (lim homeodomain), Acj6, and drifter (Pou domains) [32], [33]. In vertebrates, POU domain, Lim homeodomain and other transcription factor families have undergone large gene expansions, in parallel with the increased diversity and complexity of neuronal cell types [31], [34], [35].

We are addressing the mechanisms of RGC type specification building on the combinatorial expression of the three Brn3 POU domain transcription factors in these neurons. Brn3b is expressed by most RGCs coincident with their birth (Embryonic day E10.5–E11.5), followed by Brn3a and Brn3c expression beginning with E12.5 [36]–[39]. Combinatorial expression of Brn3s has been described for projection neurons of the somatosensory, proprioceptive, auditory and vestibular but not olfactory or gustatory systems [36]–[40]. Knock-out phenotypes of the three Brn3 genes suggest major roles in RGC (*Brn3b*), Dorsal Root Ganglia (DRG) (*Brn3a*) and auditory and vestibular hair cell (*Brn3c*) generation, however their role in cell type specification in these sensory pathways is still unclear [15], [40]–[47].

In adult mice, Brn3a and Brn3b are co-expressed in 6 RGC cell types, and *Brn3b^−/−^* retinas have severe defects in RGC specification and significant loss of Brn3a [15], [41], [42], [47], suggesting that Brn3b regulates Brn3a-RGCs survival, and/or Brn3a transcription. Since Brn3s exhibit significant overlap in sequence specificity of DNA regulatory target sites [36], [39], and Brn3a can substitute for Brn3b when knocked in at the Brn3b locus (*Brn3b^Brn3a/−^*) [48] Brn3a and Brn3b were thought to have redundant functions in RGC survival. Using our conditional reporter knock-in strategy, in which the endogenous gene is replaced with the reporter Alkaline Phosphatase (AP), we found that RGCs surviving Brn3b ablation (Brn3b^AP/−^ RGCs) exhibit major defects in axon formation and some dendritic arbor defects [15], [47], [49]. However, Brn3a protein expression is found in similar fractions (0.8) of either Brn3b^AP/+^ or Brn3b^AP/−^ RGCs, while its overall expression in the Ganglion Cell Layer (GCL) decreases from about 40% in wild type to about 10–15% in *Brn3b^−/−^* or *Brn3b^AP/−^* retinas [15], [50], inferring that loss of Brn3a in *Brn3b^−/−^* retinas is more likely due to overall Brn3a-RGC number reduction. It is however unknown if these Brn3b dependent Brn3a-RGCs belong to specific cell types, and if Brn3b independent RGCs use Brn3a for their survival.

Loss of Brn3a in Brn3a^AP/−^ RGCs results in a bias towards bistratified dendritic arbor morphologies [15], [47], but it is still unresolved whether this bias is a result of cell death, cell fate alteration and or specific morphological defects of Brn3a null RGCs.

Retinal Brn3c expression is restricted to a small percentage of RGCs (10–20%) and is distributed amongst three cell types, exhibiting either “ON”, “OFF”, or “bistratified” dendritic arbor morphologies [42], [47], but no significant defects were identified in either *Brn3c^−/−^* or *Brn3c^AP/−^* RGCs. However, numbers of Brn3c positive cells are reduced in *Brn3b^−/−^* retinas and *Brn3b^−/−^; Brn3c^−/−^* retinas have more severe RGC losses when compared to *Brn3b^−/−^* retinas. [42], [47], [51]. It is unknown how the three Brn3c^AP^ RGC types are affected by Brn3b ablation.

Collectively, these results suggest that Brn3s may have distinct roles in specification of individual RGC types, but raise a number of questions. Are Brn3 genes redundant with respect to RGC type development? Are the functions of the three transcription factors restricted to the RGC types that express them in the adult? What are the specific roles of Brn3s in each RGC cell type? Do their functions depend on each other? Are they collectively sufficient to explain the cell type and feature diversity in RGCs? In this study we address these questions by studying the effects of Brn3 single and double knock-outs on the generation of RGC types revealed with our conditional reporter alleles. We find that Brn3b is also required for the development of some of the Brn3a and Brn3c positive RGC that do not express Brn3b in the adult. We also establish that Brn3a is required for the development of a subpopulation of monostratified RGCs, and is sufficient to promote RGC survival in the absence of Brn3b. We also report that a small number of Melanopsin positive RGCs can survive in the absence of all three Brn3 genes, but that Melanopsin expression depends on the transcription factor Isl1. These findings expand our knowledge of the transcriptional code regulating RGC type diversity.

## Materials and Methods

### Mouse lines

We previously reported conditional knock-in reporter Brn3 alleles (*Brn3^CKOAP^*), having the following configuration: a loxP site is inserted in the 5′UTR, upstream of the initiator ATG; three repeats of the SV40 early region transcription terminator are added to the 3′UTR downstream (3′) of the translation termination codon, followed by a second loxP site and the coding region of human placental alkaline phosphatase (AP) Upon Cre mediated excision, the endogenous Brn3 gene is removed and replaced by AP, which marks the neurons that normally express the gene (Brn3b^AP^). If the Brn3 allele on the sister chromosome is normal, this results in a Brn3 heterozygote cell (Brn3b^AP/+^), while pairing with a null allele on the sister chromosome results in a complete Brn3 null cell (Brn3b^AP/−^). For more details see [15], [40], [47], [52]. In this study, we generate double knock-out (KO) mice by crossing a conventional null allele of one Brn3 transcription factor (*Brn3x^−^*) with a conditional knock-in reporter allele of a second Brn3 transcription factor (*Brn3y^CKOAP^*). We then analyze the effect of losing the first Brn3 transcription factor (*Brn3x^−/−^*) on the RGC types expressing the second (*Brn3y^AP/+^*). In addition, we analyze the effects of losing both Brn3 factors on surviving double knock-out RGCs (*Brn3x^−/−^*; *Brn3y^AP/−^*), and the overall RGC population.

Other used mouse lines are as follows: (a) Cre lines – Pax6α: Cre [53], Six3: Cre [54] (b) Brn3 conventional knock-out alleles: – *Brn3a^−^* [44] *Brn3b^−^* [42] and *Brn3c^−^* [45], (c) Isl1 conditional knock-out allele: *Isl1^CKO^* [28]. All mice used were adults of mixed C57Bl6/SV129 background, following procedures approved by the National Eye Institute Animal Care and Use Committee (ACUC).

To generate pair wise double-knockout lines, the following crosses were performed (numbers in parentheses refer to the mice analyzed by AP histochemistry):

(1) males *Pax6*α: Cre; *Brn3b ^+/−^; Brn3a ^+/−^* x females *Brn3b ^−/−^; Brn3a ^CKOAP/CKOAP^*, resulting in the following 4 possible offspring: (1.1) *Pax6*α: Cre; *Brn3b ^+/−^; Brn3a ^CKOAP/+^* (n = 5), (1.2) *Pax6*α: Cre; *Brn3b ^+/−^; Brn3a ^CKOAP/−^* (n = 5), (1.3) *Pax6*α: Cre; *Brn3b ^−/−^; Brn3a ^CKOAP/+^* (n = 3), (1.4) *Pax6*α: Cre; *Brn3b ^−/−^; Brn3a ^CKOAP/−^* (n = 4).

(2) males *Pax6*α: Cre; *Brn3b ^+/−^; Brn3c ^+/−^* x females *Brn3b ^−/−^; Brn3c ^CKOAP/CKOAP^* resulting in the following 4 possible offspring: (2.1) *Pax6*α: Cre; *Brn3b ^+/−^; Brn3c ^CKOAP/+^* (n = 2), (2.2) *Pax6*α: Cre; *Brn3b ^+/−^; Brn3c ^CKOAP/−^* (n = 3), (2.3) *Pax6*α: Cre*; Brn3b ^−/−^; Brn3c ^CKOAP/+^* (n = 4), (2.4) *Pax6*α: Cre; *Brn3b ^−/−^; Brn3c ^CKOAP/−^* (n = 3).

(3) males *Pax6*α: Cre; *Brn3c ^+/−^; Brn3a ^+/−^* x females *Brn3c ^−/−^; Brn3a ^CKOAP/CKOAP^*, resulting in the following 4 possible offspring: (3.1) *Pax6*α: Cre; *Brn3c ^+/−^; Brn3a ^CKOAP/+^* (n = 3), (3.2) *Pax6*α: Cre; *Brn3c ^+/−^; Brn3a ^CKOAP/−^* (n = 4), (3.3) *Pax6*α: Cre; *Brn3c ^−/−^; Brn3a ^CKOAP/+^* (n = 4), (3.4) *Pax6*α: Cre; *Brn3c ^−/−^; Brn3a ^CKOAP/−^* (n = 3),.

### Histology and Immunohistochemistry\

Retina whole mounts and brain sections were stained, processed, and imaged, and RGC dendrites and axons were traced and quantified as previously described [55], [56]). In brief, mice were anesthetized, perfusion fixed with 4% Paraformaldehyde (PFA), retinas were dissected and flat mounted, and 150 μm thick coronal brain sections were generated using a vibratome. Low resolution images of brain sections were acquired on a Zeiss Stemi SV 11 Stereomicroscope, using a Zeiss Axiocam MRC color camera. High resolution images were captured on a Zeiss Imager. Z2 fitted with an Apotome for fluorescent imaging and Axiovision software. For immunofluorescence, retina sections were processed and immunostained as previously described (Badea et al 2009a). Briefly, retinas were immersion fixed for 30 minutes with 2% paraformaldehyde, cryoprotected in OCT, and sectioned at 14 μm thickness on a cryostat. For each genotype, retinas from three distinct animals were imbedded collectively in the same OCT block, and all staining procedures were carried out collectively on these slides. Pie charts and box whisker plots represent pooled cell counts and averages from 3 or more sections from 2–3 mice/genotype. Retrograde tracing of RGC axons. For retrograde axon tracing, eyes were enucleated, fixed for 1hour in 4% PFA, Di-I (a lipohilic dye) crystals were placed on the stumps of the optic nerve, and allowed to settle for 20 minutes at room temperature. Excess DI-I was washed off with PBS, and then eyes were incubated overnight at 37 C, and then kept at 4 C until imaging. Retinas were flat mounted and imaged as above.

### Antibodies

Please see table 1 for source and working dilutions of all primary antibodies used in this study. Donkey secondary antibodies coupled with Alexa dyes, and DiI were from Molecular Probes/Life technologies (Carlsbad, CA). Neurofilament Light Chain (NFL): When used for western blots, 1∶1000 dilution detects NFL on 10 μg of mouse brain lysates. In immunofluorescence experiments, it is restricted to RGCs [57]. Melanopsin (Opn4): This antibody is specific for mouse Melanopsin and does not recognize opsins from other species. It specifically labels Opn4 positive RGCs and does not stain retinas from Opn4 −/− mice [58], [59]. Islet 1 (Isl1): Monoclonal antibody against islet-1 recognizes a 36-kDa protein on Western blots [data sheet from the Developmental Studies Hybridoma Bank). In retinal sections, the antibody identifies ganglion cells, bipolar cells and ChAT amacrine cells. It does not stain Isl1 – KO retinas [28], [29], [60]. Choline Acetyl transferase (ChAT): This widely used goat polyclonal antiChAT recognizes a 68–70 kD band on western blot, and exhibits the characteirsitic lamination pattern of cholinergic starburst amacrine cells in retinal sections. Alkaline Phosphatase (AP): This is an affinity purified mouse monoclonal antibody (E6 clone) recognizing only the human placental Alkaline Phosphatase isoform. It has been used extensively to label transgenic lines containing the AP reporter, and faithfully reproduces the expression pattern observed by AP histochemistry in the same type of tissue. [15], [40], [61]. Goat anti-Brn3: Brn-3b is an affinity purified goat polyclonal antibody raised against a peptide mapping within an internal region of Brn-3b of human origin. specifically recognizes three bands in western blot corresponding to the three Brn3 family members: Brn3a at 53 kDa, Brn3b at 51 kDa, and Brn3c at 42 kDa (manufacturer' information). In our hands, this antibody labels specifically cell bodies in the GCL, and can be used as a pan-Brn3 antibody, as we find that it labels GCL layer bodies from all single Brn3 KO retinas, but not the Brn3a – Brn3b DKO retinas. It also is used as a pan-Brn3 antibody in [62]. Rabbit polyclonal anti-Brn3a Brn3b and Brn3c: Rabbit polyclonal anti-Brn3a, anti-Brn3b anti-Brn3c antisera were previously described [36], [38]. They were raised against polypeptides from human Brn3a, Brn3b and Brn3c (see table) fused to T7 bacteriophage protein 10 (via the pGEMEX system). The antisera were affinity purified using the same respective peptides fused with Maltose Binding Protein (NEB). They do not cross react on western blots for the three proteins, and do not stain retinas from the respective KO mice. Calcium Binding protein 5 (CaBP5): Rabbit Polyclonal anti-Calcium Binding Protein 5 (CaBP5) was a generous gift of F. Haeseleer and K. Palcewsky. It specifically recognizes recombinant CaBP5 (18 kD) but not other CaBPs, [63].

**Table 1:** Antibodies used in this study.

Antigen	Immunogen	Manufacturer, Species, type Catalogue number	Dilution
Neurofilament Light Chain –66 kD	Purified Porcine NFL	Chemicon – Millipore Rabbit polyclonal, AB9568	1:500
Melanopsin	15 N terminal aa of mouse Opn4	Advanced Targeting Systems, Rabbit Polyclonal, AB-N38	1:2000
Isl-1	C Terminal domain of Isl1	Developmental Studies Hybridoma Bank, mouse monoclonal, clone 39.4D5	1:20
Choline Acetyl Transferase	human placental enzyme	Chemicon – Millipore Goat polyclonal, AB144P	1:100
Alkaline Phosphatase	Human placental alkaline phosphatase	VIB Gent, Mouse monoclonal, E6 clone	1:100
Brn3	raised against a peptide mapping at the C-terminus of Brn-3b of human origin	Santa Cruz, Goat Polyclonal, SC6026	1:50
Brn3a	human Brn3a aa 186–224– protein10 fusion (pGEMEX)	Rabbit polyclonal, generated in house, see Xiang et al 1995	1:10
Brn3b	human Brn3b aa 184–252– protein10 fusion (pGEMEX)	Rabbit polyclonal, previously described, see Xiang et al 1995 1993	1:20
Brn3c	human Brn3c aa 110–180– protein10 fusion (pGEMEX)	Rabbit polyclonal, previously described, see Xiang et al 1995 1993	1:10
Calcium Binding Protein 5	Bacterially expressed CaBP5	Rabbit polyclonal	1:400

### Imaging single cell dendritic arbors and image processing

High resolution images were captured on a Zeiss Imager. Z1 fitted with an Apotome for fluorescent imaging and Axiovision software. RGC dendritic arbors from whole mount retinas were imaged with a black and white Axiocam camera using DIC/Nomarsky optics, and neuronal arbors were reconstructed using Neuromantic neuronal tracing freeware (Darren Myat, http://www.reading.ac.uk/neuromantic), and imported and visualized using a Matlab (Mathworks) script developed in house by Friedrich Kretschmer, and which is provided as Text S1.

### Statistical analysis

Replicates are all biological. All statistical comparisons were based on assumption of draws from normal distributions. T tests were two tailed. All n, averages and standard deviations are reported in Figure legends. Explanation of Box Whisker plots: the tops and bottoms of each “Box”are the 25th and 75th percentiles of the samples, respectively. The distances between the tops and bottoms are the interquartile ranges. The line in the middle of each box is the sample median. Whiskers are drawn from the ends of the interquartile ranges to the furthest observations within the whisker length (the adjacent values).

## Results

### Genetic strategy for generating Brn3 double knock-out RGCs

**Figure 1:**
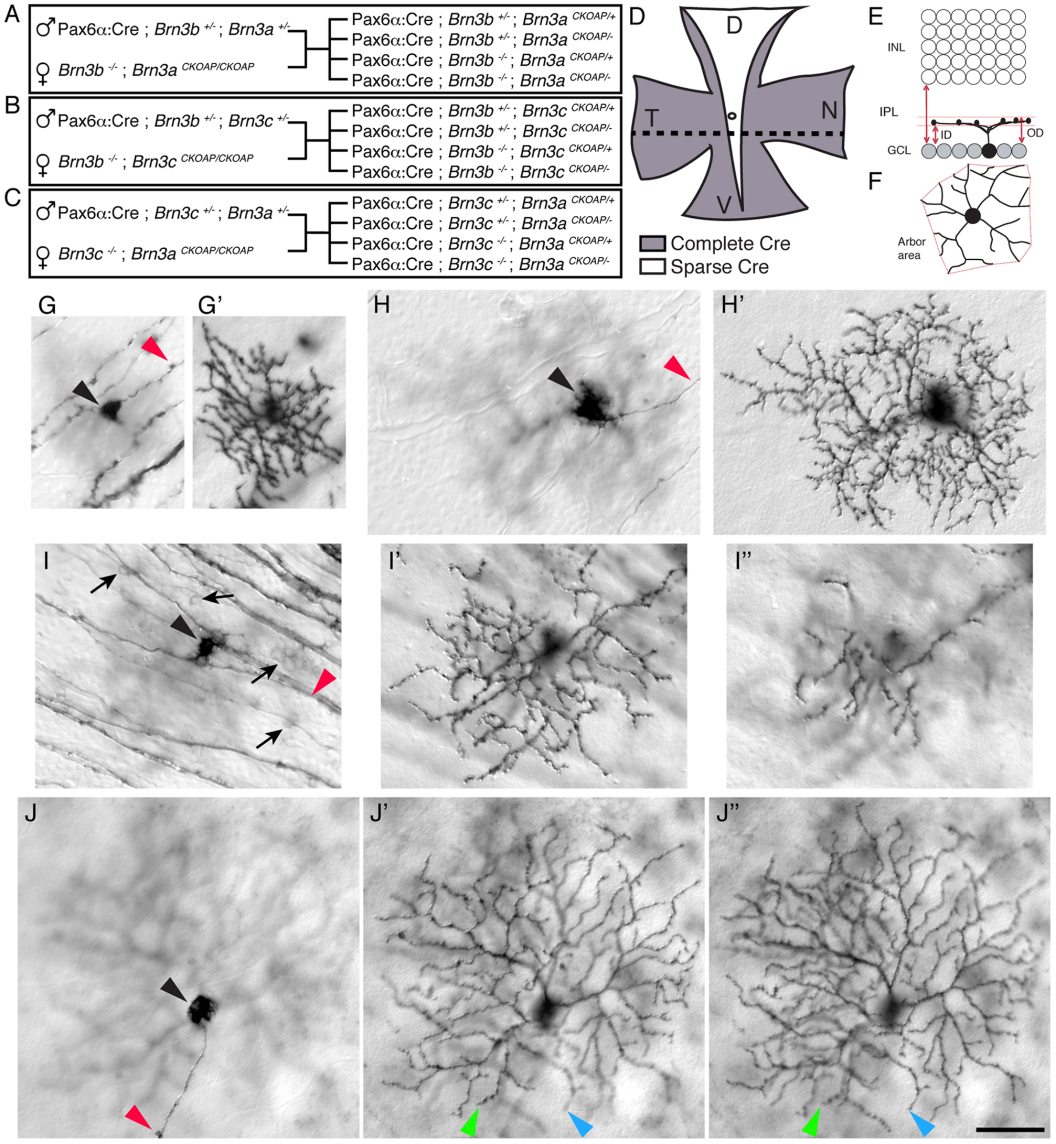
Generation and strategy of analysis of Brn3 double knock-out RGCs and controls. A–C, Crosses resulting in either *Brn3b^−/−^; Brn3a^CKOAP/−^* (A), *Brn3b^−/−^; Brn3c^CKOAP/−^* (B), or *Brn3c^−/−^; Brn3a^CKOAP/−^* (C) retinas, and appropriate double heterozygote and single Brn3 knock-out controls. For each cross the parents are indicated on the left, and the four possible types of offspring on the right. D, Diagram of retina flat mount preparation of a Pax6α: Cre mouse. In the gray shaded temporal (T), and nasal (N) areas, Cre protein is expressed in all retinal precursors, beginning with embryonic day 10, before the first RGCs start to express any Brn3. In the dorsal to ventral (D–V) oriented white sector, Cre is expressed only in a minority of cells, allowing for visualization of single cell morphologies, as shown in G – J'', and Figures 2, 3 and 4. Stippled line indicates orientation of sections for Indirect Immunofluorescence (IIF), presented in Figures 5, 7 and 9. E–F, Parameters used for single cell analysis of Brn3^AP^ RGCs, described in Figures 6,8. E, Extent of dendritic arbor lamination within the Inner Plexyform Layer (IPL) is quantified as distance between the Ganglion Cell Layer (GCL) and the inner (ID) and outer (OD) limits of the dendritic arbor and normalized to the thickness of the IPL, to yield normalized inner (ID/IPL) and outer (OD/IPL) distance. F, Dendritic arbor area in the flat mount perspective. G–J'', Nomarsky (DIC) images of individual Brn3^AP^ RGCs from the area of sparse Cre recombination. Sequential pictures in the focal plane of the cell body and axon (G, H, I, J) or the single (G', H') or double dendritic arbors (I', I'', J', J'') are shown. Genoypes are: Pax6α: Cre; *Brn3b^+/+^; Brn3a^CKOAP/+^* (G–G'), Pax6α: Cre; *Brn3b^−/−^; Brn3c^CKOAP/+^* (H–H'), Pax6α: Cre; *Brn3b^+/+^; Brn3c^CKOAP/+^* (I-I'-I''), Pax6α: Cre; *Brn3b^−/−^; Brn3a^CKOAP/−^* (J-J'-J''). Arrowheads point at cell bodies (black), axon (red), and dendrites in two different focal planes (green and blue, in J' – J''). Arrows in I point at faintly stained Brn3c^AP^ RGC cell bodies. Scale bar in J'' = 50 μm.

**Figure 2:**
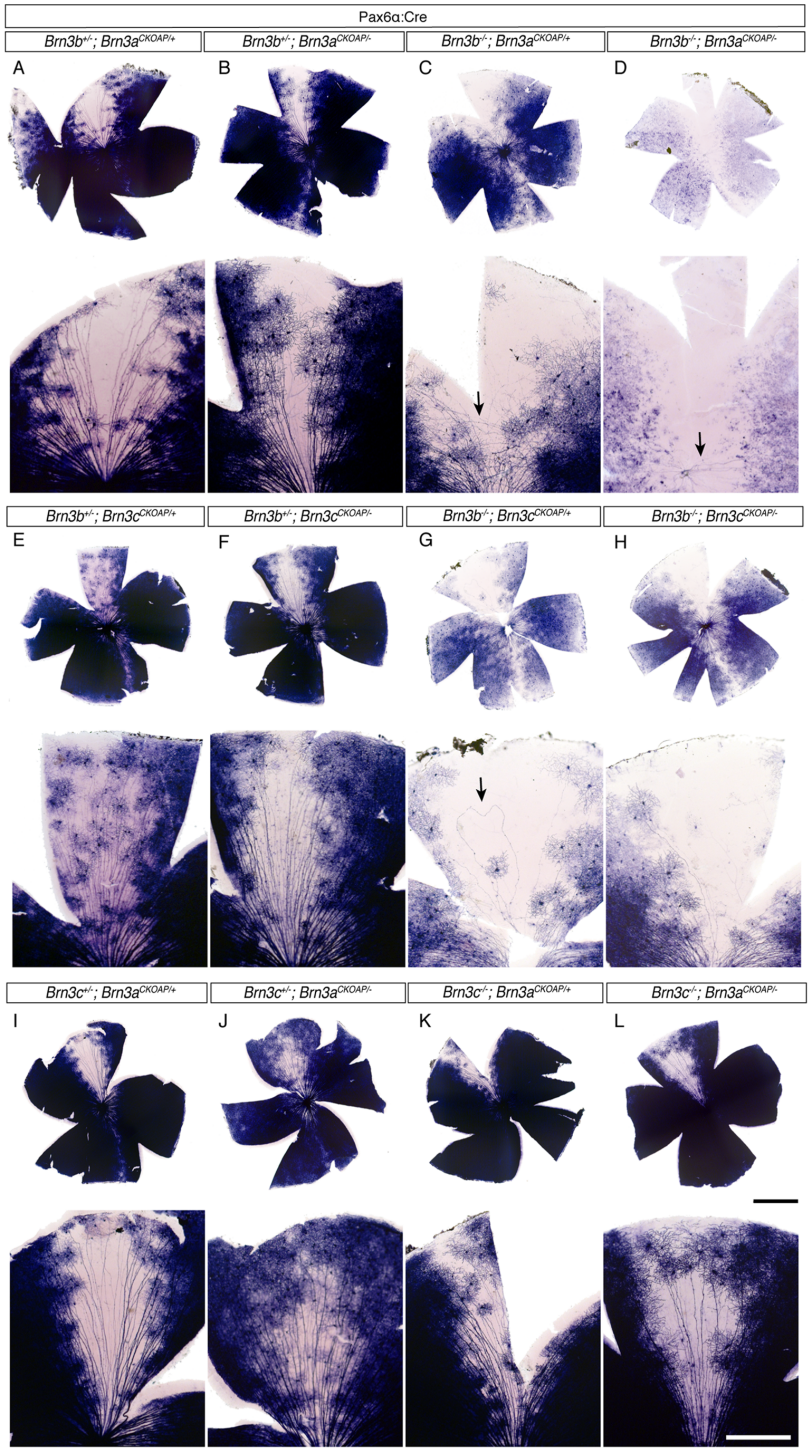
Distinct degrees of RGC loss in mice mutant for either one or two of the three Brn3 transcription factors. For each genotype (A–L), AP histochemistry of retina flat mount preparations (top) and insets covering the area of sparse recombination (bottom) are shown. AP staining is derived from Brn3a^AP^ RGCs in retinas A–D and I–L, and from Brn3c^AP^ RGCs in E–H. Genotypes and numbers of mice analyzed (n) are as follows: (A) – *Pax6*α: Cre; *Brn3b^+/−^; Brn3a^CKOAP/+^*, n = 5 (B) – Pax6α: Cre; *Brn3b^+/−^; Brn3a^CKOAP/−^*, n = 5 (C) – Pax6α: Cre; *Brn3b^−/−^; Brn3a^CKOAP/+^*, n = 3 (D) – Pax6α: Cre; *Brn3b^−/−^; Brn3a^CKOAP/−^*, n = 4 (E) – Pax6α: Cre; *Brn3b^+/−^; Brn3c^CKOAP/+^*, n = 2 (F) – Pax6α: Cre; *Brn3b^+/−^; Brn3c^CKOAP/−^*, n = 3 (G) – Pax6α: Cre*; Brn3b^−/−^; Brn3c^CKOAP/+^*, n = 4 (H) – Pax6α: Cre; *Brn3b^−/−^; Brn3c^CKOAP/−^*, n = 5 (I) – Pax6α: Cre; *Brn3c^+/−^; Brn3a^CKOAP/+^*, n = 3 (J) – Pax6α: Cre; *Brn3c^+/−^; Brn3a^CKOAP/−^*, n = 4 (K) – Pax6α: Cre*; Brn3c^−/−^; Brn3a^CKOAP/+^*, n = 4 (L) – Pax6α: Cre; *Brn3b^−/−^; Brn3a^CKOAP/−^*, n = 3. Significant numbers of Brn3a^AP^ RGCs are present in double heterozygote (A), Brn3a (B) and Brn3b (C) single knockout retinas, but are almost completely absent from Brn3a – Brn3b DKO retinas (D). Arrow in C points at tangentially arranged axon arbors. Arrow in D points at the few remaining axons tracking to optic disc. Brn3b^−/−^ retinas exhibit dramatic losses of Brn3c^AP^ RGCs (G and H), however additional removal of Brn3c (compare H to G) does not result in a larger loss of Brn3c^AP^ RGCs. Note that a large number of weakly AP positive RGC cell bodies can be seen in the inset in F. Arrow in G points at an abnormal axon tracking through the sparse region before returning towards the optic disc. Axon guidance defects as seen in C and G are present in all *Brn3b^−/−^* retinas analyzed in this study, and are reminiscent of the ones previously published. No distinct effects on Brn3a^AP^ RGCs can be observed when Brn3c is deleted either alone (J) or in combination with Brn3a (L). Scale bars in L are 1 mm (black) for retina and 250 μm (white) for inset.

**Figure 3:**
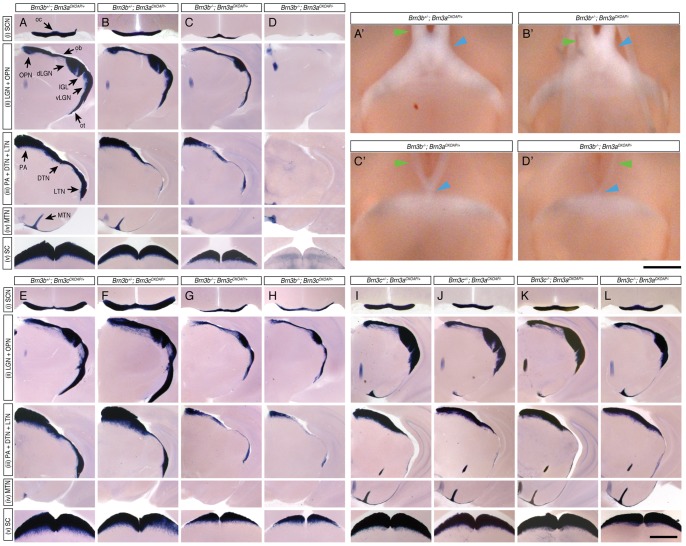
RGC axon projections to retinorecipient areas of the brain are differentially affected by loss of pairwise combinations of two Brn3 transcription factors. Columns A–L represent coronal sections through the brains of mice with identical genotypes to the ones presented in Figure 1 A–L. For each genotype, trajectories of Brn3^AP^ RGCs through five distinct stations of the optic nerve and tract are shown: (i) optic chiasm (oc) and Suprachiasmatic Nucleus (SCN), (ii) optic tract (ot), ventral Lateral Geniculate Nucleus (vLGN), Intergeniculate Leaflet (IGL), dorsal Lateral Geniculate Nucleus (dLGN), optic brachium (ob), Olivary Pretectal Nucleus (OPN), (iii) Caudal Pretectal Area (PA), Dorsal Terminal Nucleus (DTN), and Lateral Terminal Nucleus (LTN), (iv) Medial Terminal Nucleus (MTN), and (v) Superior Colliculus (SC). Nuclei are in capital letters, and axon tracts are in lower case. Consistent with RGC losses in the retina, only combined loss of Brn3a and Brn3b results in a complete removal of RGC axons from all portions of the optic path and retinorecipient nuclei (A–D). A'– D', Ventral views of unstained whole mouse brains with same genotypes as A–D. Note the reductions in optic nerve (green arrow) diameters and thickness and position of the optic chiasm (blue arrow) in Brn3b KO (C') and Brn3a-Brn3b DKO (D') mice. Brn3c^AP^ RGC projections to all targets (dLGN and SC) are equally reduced in the absence of Brn3b (G) or combined deletion of Brn3b and Brn3c (H). Similar data were obtained from all mice of all genotypes as described all in Fig. 2. Scale bar in L = 1 mm.

We investigated the genetic interactions between pairs of the three Brn3 transcription factors, by crossing conventional null (KO or -) and conditional Knock-in reporter (CKOAP, AP after recombination) alleles (Fig. 1A–C, materials and methods). In each of the three crosses, one Brn3 gene is present either as a heterozygote or a homozygote null over the entire retinal surface. The other Brn3 gene is conditionally removed using the Pax6α: Cre transgene [53] expressed specifically in the retina beginning with E9.5-E10.5, prior to the expression of the Brn3 genes in RGCs. Over most of the retinal surface, Pax6α: Cre expression is fully penetrant, resulting in complete recombination (Fig. 1D, shaded area). In a dorso-ventrally oriented retinal sector, Cre is expressed in a small number of cells, resulting in sparse recombination and cell labeling (Fig. 1D, white area). In either case, RGCs are labeled by the *Brn3^AP^* allele, in which the Brn3 open reading frame has been replaced with the AP reporter in a Cre dependent manner [15]. Therefore, results using AP staining refer to this subpopulation, which will contain either Brn3^AP/+^ (heterozygote) or Brn3^AP/−^ (homozygote null) labeled RGCs. As an example, the cross described in Figure 1A will result in 4 distinct outcomes containing RGCs with the following genotypes: (i) *Brn3b^+/−^; Brn3a^AP/+^*, (ii) *Brn3b^+/−^; Brn3a^AP/−^*, (iii) *Brn3b^−/−^; Brn3a^AP/+^*, and (iv) *Brn3b^−/−^; Brn3a^AP/−^*. Since all described experiments are using Pax6α: Cre as a source of Cre recombinase, we will omit it from the genotype labels. No significant additive effects on RGC development were noted for the Brn3c-Brn3a DKO retinas or RGC brain projections (Fig.2 I–L and Fig. 3I–L) and they were not further investigated.

### Brn3a and Brn3b mutations exhibit cumulative effects with respect to RGC loss

**Figure 4:**
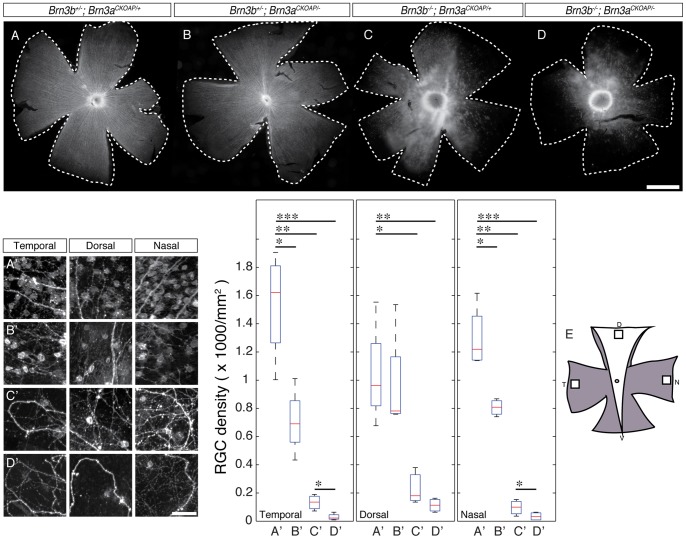
Total RGC numbers are significantly reduced by combined ablation of Brn3a and Brn3b. Overall numbers of RGCs were estimated by retrograde labeling of cell bodies with DiI crystals placed on the optic nerve stump, about 0.5(materials and methods). A–D, Flat mounts from (A) – *Brn3b ^+/−^; Brn3a ^CKOAP/+^*, n = 4, (B) – *Brn3b ^+/−^; Brn3a ^CKOAP/−^*, n = 4 (C) – *Brn3b ^−/−^; Brn3a ^CKOAP/+^*, n = 3 (D) – *Brn3b ^−/−^; Brn3a ^CKOAP/−^*, n = 4 retinas were oriented as schematized in (E). E, To standardize cell counts, temporal (T), dorsal (D), and nasal (N) 1 mm^2^ square images were taken, aligned to the outer edges of the tissue. White sector denotes the area of the retina in which only the Brn3b gene is ablated, whereas gray zones coincide with the expression domain of the Pax6α: Cre transgene, and hence both Brn3b and Brn3a gene copy numbers are being manipulated. A'– D', Insets, left panel, and box whisker plots, right panel, from the three quadrants shown in (E), demonstrate incremental loss of RGC cell bodies correlating with gene loss of function for Brn3a (B'), Brn3b (C') or both (D'). The box-whiskers plots for each of the three locations (temporal, dorsal, nasal) and four genotypes (A'–D') are shown. Note that, in *Brn3b^−/−^* only retinas (C'), total RGC numbers are dramatically reduced in all three analyzed quadrants, whereas in *Brn3a^CKOAP/−^* retinas (B'), RGC numbers are significantly reduced only in the temporal and nasal quadrants, where the Pax6α: Cre transgene is expressed and hence both copies of the Brn3a gene are removed in a nearly complete fashion. Whereas Brn3b ablation has the most dramatic effect on RGC loss (compare A' to C'), additional ablation of Brn3a further reduces the total number of RGCs (compare C' to D'). Horizontal bars in panels A'–D' denote pairs tested for significance by t tests (significance levels ^*^p<0.05, ^**^p<0.005, ^**^p<0.001). RGC density averages, expressed as cells/mm^2^, are: for the temporal quadrants A' = 1537.16, B' = 706.51, C' = 132.42 and D' = 29.34; for the dorsal quadrants A' = 1038.32, B' = 963.83, C' = 231.74 and D' = 112.86; for the nasal quadrants A' = 1297.90, B' = 805.82, C' = 96.31 and D' = 33.86. Scale bars are 1 mm in d and 50 μm in D'.

**Figure 5:**
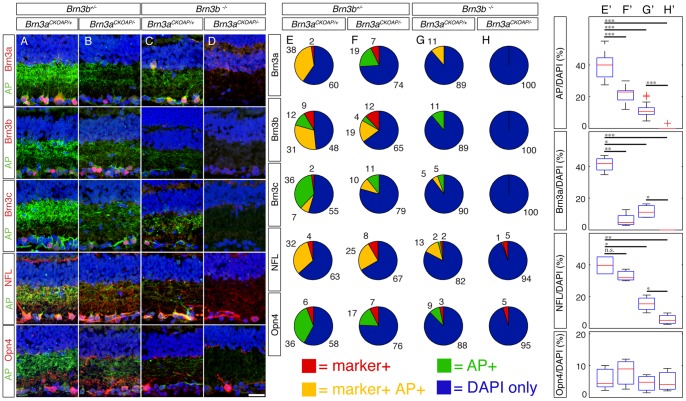
Cumulative RGC loss in Brn3a – Brn3b double KO retinas is confirmed by drastic reduction in other general RGC markers. Retinal sections cut in a naso-temporal plane, as shown in Figure 1D, where stained with antibodies against AP and a variety of markers. Imaged and quantitated regions fall within the Pax6α: Cre full recombination domain. Genotypes indicated at the top are the same as the ones seen in Fig. 2A–D. A–D, examples of retinas from the four genotypes stained with antibodies against AP and various RGC markers, as quantitated in E–H. E–H, Pie charts represent quantitations of Brn3a^AP^ RGCs stained for AP in conjunction with either antibodies against the Brn3a (row 1), Brn3b (row 2) or Brn3c (row3) proteins or the RGC markers Neurofilament Light chain (NFL, row 4) and Melanopsin (Opn4, row 5). To normalize the observed frequencies of AP only (green), marker only (red), or double positive cells (yellow), the number of DAPI only cells in the GCL is included (blue). Numbers next to the pie sectors are percent of total. E'–H', box whisker plots for relative densities of four RGC markers in the GCL, for the same experiments and genotypes presented in E–H. For Brn3a, NFL and Opn4, 4–7 sections from 2–3 mice were analyzed for each marker and genotype; for AP, 16–21 sections have been quantitated for each genotype. Marker density averages, expressed as Marker/DAPI cells in GCL, are: AP: E' = 39.08%, F' = 21.21%, G' = 11.54% and H' = 0.20%; Brn3a: E' = 41.31%, F' = 6.2%, G' = 11.62% and H' = 0.00%; NFL: E' = 39.64%, F' = 33.04%, G' = 15.4%, and H' = 5.55%; Opn4: E' = 5.84%, F' = 7.83%, G' = 4.43%, and H' = 5.04% (significance levels ^*^p<0.05, ^**^p<0.005, ^**^p<0.001). Note that retinas lacking both Brn3a and Brn3b are completely devoid of Brn3a^AP^ RGCs, Brn3a, Brn3b and Brn3c, but preserve a few NFL and Opn4 positive RGCs, representing each about 5% of DAPI positive cells in the GCL (a–d, rows 4 and 5). Scale bar in D is 25 μm.

**Figure 6:**
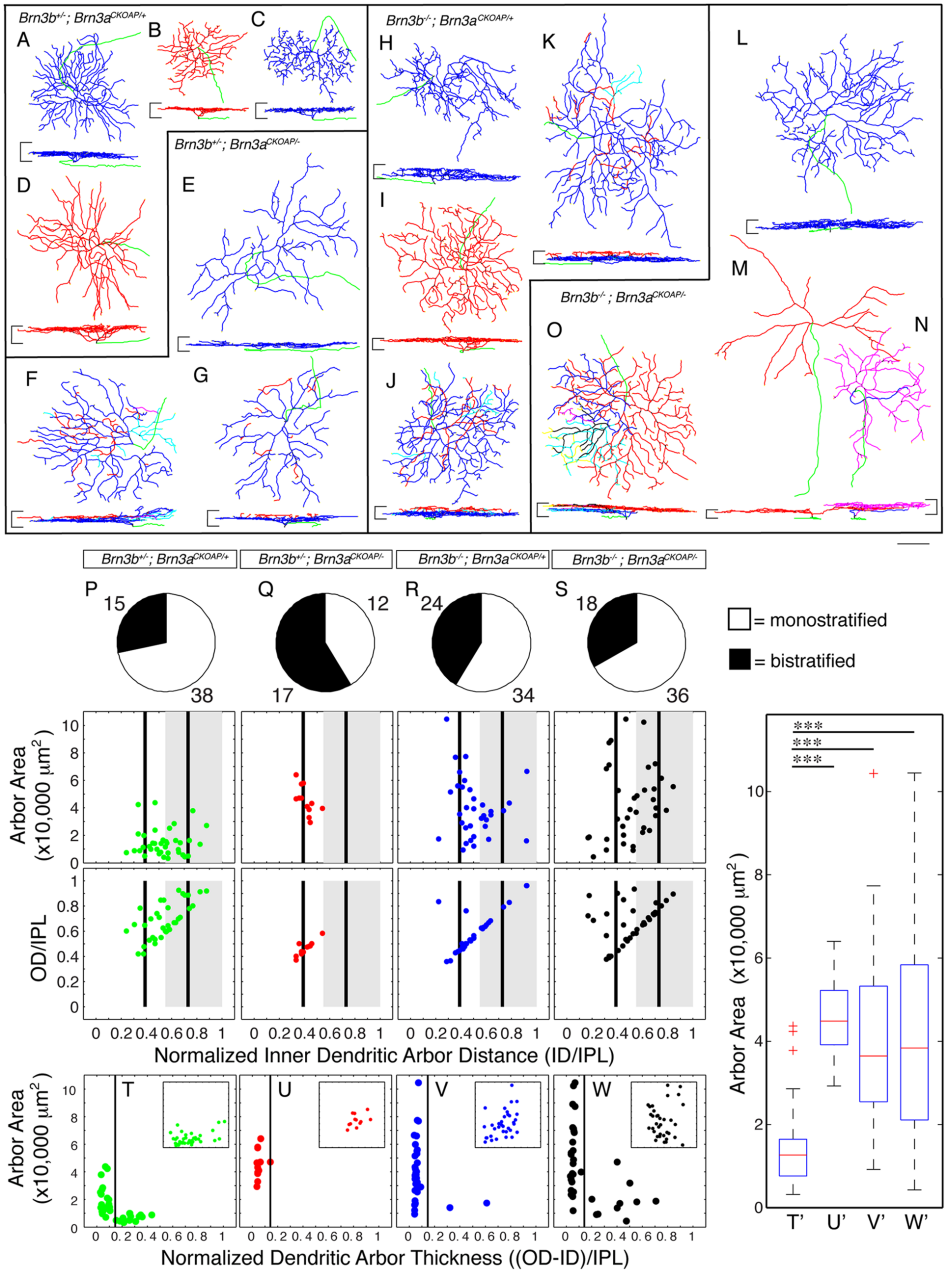
Distinct morphological defects in Brn3a^AP^ RGCs missing either Brn3a, Brn3b or both transcription factors. A–O, morphological reconstructions of Brn3a^AP^ RGCs from the area of sparse recombination in retinas of genotypes identical to Fig. 2A–D (for numbers of mice for each genotype, see Fig. 2). For each indicated genotype, several examples are shown, surrounded by black outlines. For each individual RGC, *en face* (top) and vertical view (bottom) are shown. Stratification levels for each cell can be compared to the GCL and INL limits of the IPL, shown as horizontal black lines at the sides of the vertical view. Axons are shown in green, ON stratifying dendrites in blue and OFF stratifying dendrites in red. In bistratified RGCs, cyan, magenta, black and yellow are used for “recurrent dendrites” which originate from the OFF plexus, and cross between to the ON and then OFF plexuses, several times. Scale bar is 50 μm. P–W, Quantitations of morphological parameters for Brn3a^AP^ RGCs. For each indicated genotype, top panels are pie charts of bistratified (blue) vs. monostratified (red) RGCs. For each genotype, the number of cells of each type is indicated. Middle and bottom panels contain morphological parameters for monostratified RGCs. Middle panels show scatter plots of vitread dendritic arbor lamination limit (ID/IPL, x axis) vs. dendritic arbor area (y axis) and bottom panels vitread vs sclerad dendritic arbor lamination limits (ID/IPL, x axis vs. OD/IPL, y axis). Black vertical bars representing the stratification levels of ' 'ON'' (0.39 ID/IPL) and “FF” (0.73 ID/IPL) starburst amacrine cells, and a gray rectangle spanning 0.55–1.0 ID/IPL representing the “FF” sublamina of the IPL serve as theoretical stratification landmarks derived from literature (see Badea & Nathans 2011). T–W, the extent of lamination depth of monostratified dendritic arbors within the IPL is expressed normalized dendritic arbor thickness, and plotted against arbor area. Vertical lines at 0.15 thickness separate large, flat dendritic arbors on the left, from small and thick dendritic arbors on the right. In each panel, the insets show data for excentricity of the cell body (x axis) plotted against dendritic arbor area (y axis), showing little correlation between the two parameters. For a detailed explanation of these parameters, see Materials and Methods, Fig. 1 e, f and Badea et al, 2004, 2009 or 2011. A significant increase in dendritic arbor area, and striking depth of lamination defects can be observed in Brn3a^AP^ RGCs missing either Brn3b or both Brn3a and Brn3b. T'–W' represent box-whisker plots for dendritic arbor areas of monostratified RGCs of same genotypes as T–W. RGC dendritic arbor area averages, in μm^2^, are: T' = 14,799, U' = 45,330, V' = 39,665 and W' = 43,147 (significance ^***^p<0.001).

Using *Brn3^CKOAP^* conditional alleles, we have previously described the cell autonomous effects of either Brn3a or Brn3b loss on adult RGCs normally expressing the two transcription factors [15], [47]. Whereas in adults Brn3a and Brn3b are co-expressed in a subset of RGC types, it is possible that during development there is a larger degree of overlap between the two transcription factors. We therefore asked whether early developmental Brn3b removal would affect all Brn3a^AP^ RGC types, or just the subpopulation which are normally also Brn3b positive in the adult, by determining RGC numbers, dendritic arbors and axonal projections in *Brn3b^−/−^; Brn3a^CKOAP/+^* retinas and brains (Fig. 2, 3, 4, 5 and 6).

Indeed, in Brn3b^−/−^ retinas, Brn3a^AP/+^ RGC numbers were significantly reduced as evident from global levels of AP staining (Fig. 2C top panel) and reduced or absent axon projections to retinorecipient brain targets (Fig. 3C). These cell losses are also quantified by determining the loss of AP positive RGC numbers in IIF sections (Fig. 5C, G). Brn3a^AP^ RGC/DAPI ratios decrease from 38% in *Brn3b^+/−^; Brn3a^CKOAP/+^* to 11% in *Brn3b^−/−^; Brn3a^CKOAP/+^* retinas (Fig. 5A, C, E, G, E', G'). In contrast, losing Brn3a in *Brn3b^+/−^; Brn3a^CKOAP/−^* retinas only reduces Brn3a^AP^ RGC/DAPI ratios to 21% (Fig. 5B, F, F' and Badea 2009, Sup Fig. 1), consistent with previous data suggesting that Brn3a is not required to maintain its own expression [64]. Thus a subset of Brn3a positive RGCs does not require Brn3b either for survival or for Brn3a expression.

Combined loss of Brn3a and Brn3b (henceforth Brn3a–3b DKO – double knock-out) in *Brn3b^−/−^; Brn3a^CKOAP/−^* retinas, results in an essentially complete ablation of Brn3a^AP^ RGCs (Fig. 2D). In these retinal flat mount preparations only about 10 remaining AP positive RGC axons per retina track to the optic nerve head. The background level of AP positivity seen in the flat mount preparation is derived from misexpression of AP in other cell types like photoreceptors, amacrines, etc. – a phenomenon we have previously described (see Badea et al 2009). Although no AP positive axons were detected in the retinorecipient areas of Brn3b – Brn3a DKO mice (Fig. 3D), it is worth noting that a small number of RGCs may survive the loss of both transcription factors, since the optic nerve, carrying RGC axons to the brain, although massively reduced, is still present, as seen from ventral views of the brain showing the optic nerve and chiasm (Fig. 3A'–D'). The massive loss of RGCs is also documented in retinal sections (Brn3a^AP^ RGC/DAPI ratio  = 0.2%, Fig. 5D, H, H').

It is crucial to understand whether Brn3a^AP^ RGCs loss seen in Brn3a KO, Brn3b KO or Brn3a–3b DKO retinas is a result of reduced RGC numbers, silencing of the Brn3a^AP^ allele, or a combination of both. It is also of high interest to understand whether the surviving RGCs in each genotype can be assigned to specific cell types. To address these questions, we sought to determine the total number of RGCs in wild type, single, or double Brn3a and Brn3b knock-out retinas by DiI retrograde tracing of RGC axons from the optic nerve (Fig. 4), indirect immunofluorescence detection of other RGC markers (Fig. 5), and compared the Brn3a^AP^ RGCs dendrite morphologies in the four different genotypes (Fig. 6).

RGCs are collectively defined as the retinal neurons that send axons through the optic disk and optic nerve to the brain. We quantitated RGCs based on this definition by labeling axon-projecting cell bodies retrogradely from the optic nerve. To account for the expression pattern of the Pax6α: Cre transgenic driver, and hence the differential gene ablation of the *Brn3a^CKOAP^* allele in the distinct quadrants of the retina, mouse eyes were marked before removal from the head, RGC axons retrogradely labeled with DiI applied to the optic nerve stump, and RGC cell numbers quantified from distinct regions of the oriented retinas (Fig. 4). At the temporal and nasal locations full recombination should result in essentially complete conversion to Brn3a^AP^, while the sparse recombination in the dorsal quadrant is unlikely to significantly affect total RGC numbers. Indeed, temporal and nasal quadrants of *Brn3b^+/−^; Brn3a^CKOAP/−^* retinas do exhibit a significant (45 and 62%) loss in DiI positive RGC numbers as compared to *Brn3b^+/−^; Brn3a^CKOAP/+^* controls, whereas the dorsal quadrant is not significantly affected (92%, Fig. 4 compare B' to A'). However, total numbers of RGCs are severely reduced in all three quadrants in *Brn3b ^−/−^; Brn3a ^CKOAP/+^* retinas, from which Brn3b is lost in a global fashion (7% for temporal, 8% for nasal and 22% for dorsal quadrants, compare Fig. 4C' to A'). Combined Brn3a and Brn3b loss in the temporal and nasal quadrants of *Brn3b^−/−^; Brn3a^CKOAP/−^* retinas further reduces RGC numbers (2%), whereas the reduction in the dorsal quadrant is less sizeable (10%, Fig. 4D'). Thus, total RGC numbers drop more significantly in response to loss of Brn3b alone when compared to the loss in Brn3a^AP^ RGCs (Fig. 2 and Fig. 5), possibly by a more pronounced relative effect on Brn3a negative Brn3b^AP^ RGCs. However, the total RGC loss in the Brn3a-Brn3b DKO compared to wild type is less dramatic (2%) when compared to the loss in Brn3a^AP^ RGCs (0.2%), potentially denoting the presence of Brn3a and Brn3b independent RGCs. Therefore, this experiment supports two novel conclusions: (i) loss of Brn3a alone results in decreased overall RGC numbers; (ii) combined Brn3b and Brn3a ablation results in more severe RGC loss than either transcription factor alone. Since the cell losses assessed by this method parallel the results shown in Figures 2 and 3 by AP histochemistry, we argue that the losses of Brn3a^AP^ seen in the three mutant backgrounds are a result of progressive loss of RGC numbers, rather than silencing of the Brn3a locus.

Independent measures of RGC numbers in single Brn3a, Brn3b and combined Brn3a-Brn3b knockouts were obtained by staining sections from retinas of the four genotypes for several known general and specific RGC markers: Neurofilament light (NFL, Fig. 5 A–H, row 4, E'–H' row 3), medium (NFM) and heavy (NFH) chain, Thy-1 (data not shown), the three Brn3 transcription factors (Fig. 5A–H, rows 1–3, E'–H', row 2), and the ipRGC marker, Melanopsin (*Opn4*, [10], [13], [58], Fig. 5A–H, row 5, E'–H' row 4). Reduction in general RGC marker counts was more moderate than that observed in DiI retrograde fill experiments or by counting Brn3a^AP^ RGCs, but the cumulative effect on general RGC loss for Brn3a and Brn3b DKO was still present. Similar results were observed for Thy-1, NFM and NFH (data not shown).

Loss of Brn3a and Brn3b proteins from their respective single knock-outs is consistent with previously published data (Fig. 5A–D, Badea et al 2009 Sup Fig. 1), and complete in the nasal and temporal full recombination sectors of the Brn3a–Brn3b DKO retinas. Loss of Brn3c was not observed in retinas lacking Brn3a, with about a 50% reduction in the *Brn3b^−/−^; Brn3a^CKOAP/+^* and complete ablation in the *Brn3b^−/−^; Brn3a^CKOAP/−^* retinas. This may suggest that either Brn3a or Brn3b can support Brn3c transcription, or Brn3c RGC survival. In contrast, Melanopsin positive ipRGCs, occurring in small percentages in controls (6–7% Opn4/DAPI), are only reduced modestly in either Brn3bKO single or Brn3a–Brn3b DKO mice, consistent with previous observations that only a fraction of ipRGCs express Brn3b and may depend on it for survival, but none express Brn3a.

Collectively, these results demonstrate that both Brn3a and Brn3b are required for the generation of normal overall RGC numbers as well as specifically Brn3a^AP^ RGCs. Whereas a majority of RGCs are under the transcriptional control of either Brn3a or Brn3b, a subpopulation of RGCs, expressing NFL and/or Melanopsin but not Brn3c, and projecting axons through the optic nerve, survives ablation of both Brn3a and Brn3b.

### Morphology of most Brn3a^AP^ RGC types is affected by Brn3b or combined Brn3b–Brn3a loss

The presented data demonstrate that early developmental Brn3b loss results in a significant but incomplete loss of Brn3a^AP^ RGCs, and that ablation of this population is essentially complete when both Brn3a and Brn3b are deleted. General RGC markers are progressively and cumulatively reduced when Brn3a, Brn3b or both are ablated, but a small RGC population survives in the double KO retinas. One possible interpretation for the RGC losses seen in Brn3a and Brn3b single and DKO retinas is that the two transcription factors are required either alone or in combination for the specification and/or survival of specific RGC cell types. This view is supported by our previous results [15], [47], specifically the bias towards bistratified RGCs seen in *Brn3a^CKOAP/−^* retinas and the complete loss of MTN projecting RGCs in the *Brn3b^−/−^* or Pax6α: Cre; *Brn3a^CKOAP/−^* mice. Alternatively, each Brn3 regulates a subset of RGC features, some of which are required for survival of specific RGC types. To distinguish between these two possibilities, we measured morphological parameters of the dendritic arbors of individual Brn3a^AP^ RGCs from the sparsely recombined areas of Pax6α: Cre expression (Fig. 1D, E and Fig. 2A–D, bottom inset panels, examples shown in Fig. 1G–J''). For RGC dendritic arbors, depth of lamination within the Inner Plexiform Layer (IPL) and area in the flat mount perspective are particularly powerful parameters of cell type classification [1], [2], [4]. The IPL is a very sharply laminated structure and dendritic arbors of most described RGC types laminate very precisely, thereby presumably restricting their synaptic interactions with specific bipolar and amacrine cells. A feature common to all Brn3^AP^ RGC types is the reverse correlation of dendrite arbor thickness across the depth of the IPL and dendrite arbor area in the flat mount perspective. Dendritic arbors spanning large areas in the flat mount orientation tend to be confined to a very thin lamina in cross section, typically less than 10–15% IPL depth, whereas some types of RGCs with dendritic arbors spanning smaller areas tend to spread across 20% or more IPL depth (Fig. 6A–D, T, and Badea et al 2009, 2011). Brn3a^AP^ RGCs in the double heterozygote control retinas exhibit dendritic arbor area distributions comparable to the ones reported before (mean  = 14,799 μm^2^, Fig. 6A–D, P second row, Badea 2009 and 2011,). This distribution is significantly shifted towards larger dendritic arbors for either *Brn3b^+/−^; Brn3a^CKOAP/−^* (mean  = 45,330 μm^2^), *Brn3b^−/−^; Brn3a^CKOAP/+^* (mean  = 39,665 μm^2^) or *Brn3b^−/−^*; *Brn3a^CKOAP/−^* (mean  = 43,147 μm^2^) (Fig. 6E–N, Q–S, second row). Whereas in the case of *Brn3b^+/−^*; *Brn3a^CKOAP/−^* some of the increase in area could be explained by an eccentricity effect (peripheral RGC dendritic arbors tend to be larger than central ones, see [47], [65], this is not the case for the other two genotypes (Fig. 6T–W, insets, plotting eccentricity vs. area). Essentially, small and thick dendritic arbor RGCs are missing from all three mutant combinations. Conversely, in *Brn3b^−/−^; Brn3a^CKOAP/−^* and to a lesser degree in *Brn3b^−/−^; Brn3a^CKOAP/+^* retinas, we observe a novel type of morphology, with large and thick dendritic arbors (Fig. 6H, L, V, W). Previously, mutant Brn3b^AP^ RGCs spanning large areas and having thick arborization depths were reported in *Brn3b^CKOAP/−^* retinas, (Badea 2009 and 2011). In contrast, monostratified RGCs in *Brn3b^+/−^; Brn3a^CKOAP/−^* retinas tend to preserve their sharp lamination (Fig.6 E, U and Badea 2009 and 2011).

Given the changes in area distribution and lamination described, it is difficult to establish correspondences between wild type Brn3a^AP^ RGC types and those in single and double Brn3a and Brn3b KO retinas. However, it is clear that in Brn3b KO retinas, Brn3a^AP^ RGCs exhibit a global increase in dendritic arbor area, sometimes more than twice the size of the largest wild type Brn3a^AP^ RGCs. In addition, RGCs with small dendritic arbors laminated over more than 20% IPL depth are missing. The apparent increase in area could be the result of selective loss of RGCs with small dendritic arbors or to a compensatory increase in arbor area, attempting to completely tile a retina that has lost significant numbers of RGCs. Alternatively, surviving Brn3a^AP^ RGCs could have altered gene expression profiles that result in a systematic bias for larger dendritic arbor areas, irrespective of original cell type.

Interestingly, intraretinal axon guidance defects observed in *Brn3b^CKOAP/−^* and *Brn3b^−/−^; Brn3a^CKOAP/+^* retinas are rarely observed in the few surviving *Brn3b^−/−^; Brn3a^CKOAP/−^* RGCs (Badea 2009 and Fig. 2C and D arrowheads). The complete ablation of the MTN projection, previously described in *Brn3b^−/−^* mice and for Brn3b^AP^ RGCs in *Brn3b^CKOAP/−^* retinas is also seen with the Brn3a^AP^ RGCs in the case of *Brn3b^−/−^; Brn3a^CKOAP/+^*, most likely reflecting co-expression of Brn3a and Brn3b in this particular type of Brn3b dependent ON DS RGCs (Fig. 3C). Despite the severe losses in RGCs, lamination of known amacrine and bipolar cell neurites is not dramatically affected (data not shown), which is consistent with the view that IPL lamination is only modestly affected by RGC dendrite development [22], [23], [66].

We conclude that Brn3a^AP^ RGC type development is globally affected by either Brn3b or combined Brn3a-Brn3b loss, with specific cell types completely absent, and surviving neurons having dendritic arbors with increased areas and reduced thickness compared to wild type Brn3a RGCs. Brn3a^AP^ RGC types, as defined by characteristic dendritic arbor morphologies, are affected by Brn3b loss regardless of whether they express Brn3b in the adult or not. These results are inconsistent with a model in which Brn3b selectively controls the development of specific Brn3a^AP^ RGC types, and suggest that the role of Brn3b in RGC type specification extends beyond its expression pattern in the adult. This could mean that Brn3b has a broader expression in RGCs during development or alternatively that Brn3b RGCs can affect Brn3b negative RGCs in a non-cell autonomous fashion, through extracellular signaling or cell-cell contact.

### Brn3b is required for the development of “OFF” stratifying Brn3c^AP^ RGCs

**Figure 7:**
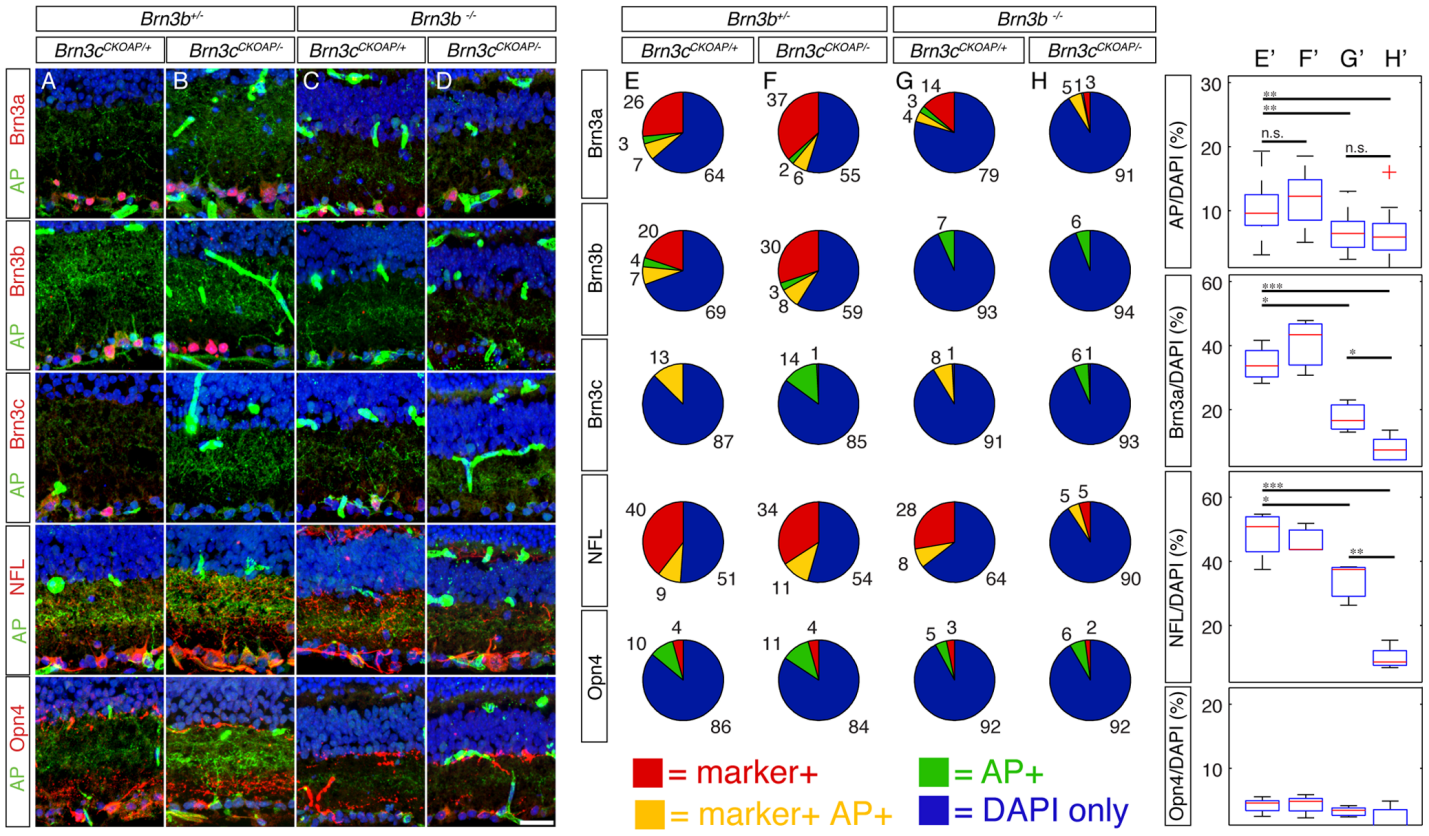
Selective RGC loss in Brn3b – Brn3c double KO retinas documented by losses of general RGC markers. Retinal sections cut in a naso-temporal plane, as shown in Figure 1D, where stained with antibodies against AP and a variety of markers. Imaged and quantitated regions fall within the Pax6α: Cre full recombination domain. Genotypes indicated at the top are the same as the ones seen in Fig. 2E–H. A–D, examples of retinas from the four genotypes stained with antibodies against AP and various RGC markers, as quantitated in E–H. E–H, Pie charts represent quantitations of Brn3c^AP^ RGCs stained for AP in conjunction with either antibodies against the Brn3a (row 1), Brn3b (row 2) or Brn3c (row3) proteins or the RGC markers Neurofilament Light chain (NFL, row 4) and Melanopsin (Opn4, row 5). To normalize the observed frequencies of AP only (green), marker only (red), or double positive cells (yellow), the number of DAPI only cells in the GCL is included (blue). Numbers next to the pie sectors are percent of total. E'–H' box whisker plots for relative densities of four RGC markers in the GCL, for the same experiments and genotypes presented in E–H. For Brn3a, NFL and Opn4, 3–7 sections from 2–3 mice were analyzed for each marker and genotype; for AP, 17–22 sections have been quantitated for each genotype. Marker density averages, expressed as Marker/DAPI cells in GCL, are: AP: E' = 10.18%, F' = 11.63%, G' = 6.57% and H' = 6.16%; Brn3a: E' = 34.34%, F' = 40.65%, G' = 17.6% and H' = 8.26%; NFL: E' = 48.47%, F' = 46.41%, G' = 34.03%, and H' = 9.86%; Opn4: E' = 4.21%, F' = 4.36%, G' = 3.3%, and H' = 1.77% (significance levels ^*^p<0.05, ^**^p<0.005, ^**^p<0.001). Note that retinas lacking Brn3b alone, or in conjunction with Brn3c have preserved AP positivity only in a restricted lamina in the centre of the IPL. Brn3a and NFL, show cumulative losses in Brn3b–Brn3c DKO retinas when compared to Brn3b single KO. Scale bar in D is 25 μm.

Being comprised of only three cell types, Brn3c^AP^ RGCs represent a simpler system for assessing Brn3 genetic interactions. We were interested in both the effects of Brn3b removal on the different Brn3c^AP^ RGC types, and the genetic interactions between the two factors (Fig. 1B). Consistent with previous results, *Brn3b^+/−^; Brn3c^CKOAP/−^* mice have normal amounts of Brn3c^AP^ RGCs in terms of overall cell numbers (Fig. 2E,F), brain nuclei targeting (Fig. 3E,F), or number of Brn3c^AP^ RGCs cell bodies in the GCL (Fig. 7A,B and E,F). However, global retinal loss of Brn3b in single Brn3b KO and Brn3b-Brn3c DKO mice results in a roughly 50% loss of Brn3c^AP^ RGCs compared to WT or single Brn3c KO (Fig. 2G, H, Fig. 3G, H, Fig. 7C, D and G, H; Fig. 7E'–H' Brn3c^AP^ RGCs/DAPI ratios  = 10% – *Brn3b^+/−^; Brn3c^CKOAP/+^*, 11% – *Brn3b^+/−^; Brn3c^CKOAP/−^*, 6% – *Brn3b^−/−^; Brn3c^CKOAP/+^*, 6% – *Brn3b^−/−^; Brn3c^CKOAP/−^*). These data do not suggest any cumulative effect between Brn3b and Brn3c mutations with respect to Brn3c^AP^ RGC survival. Therefore we assessed the effects of combined deletion of Brn3b and Brn3c on other RGC markers. Melanopsin positive cell numbers are only modestly affected by Brn3b mutation or the double KO (Fig. 7A–D, E–H, row 5 E'–H' row 4). However, numbers of NFL and Brn3a positive RGCs are reduced in combined Brn3b–Brn3c KO retinas compared to the single Brn3b KO (Fig. 7C–D, G–H, rows 1 and 4, Brn3a/DAPI and NFL/DAPI ratios  = 34.34 and 48.47% – *Brn3b^+/−^*; *Brn3c^CKOAP/+^*, 40.65 and 46.41% – *Brn3b^+/−^*; *Brn3c^CKOAP/−^*, 17.6 and 34.03% – *Brn3b^−/−^*; *Brn3c^CKOAP/+^*, 8.26 and 9.86% – *Brn3b^−/−^*; *Brn3c^CKOAP/−^*). Together, these results present the surprising conclusion that Brn3b and Brn3c mutations have cumulative effects with respect to total RGC but not Brn3c^AP^ RGC survival. One potential explanation is that Brn3c is expressed and required for RGC lineages at earlier developmental time points, whereas its expression is reduced in the adult. The other is prompted by a low level of expression of Brn3c observed with the AP reporter, but less with anti-Brn3c antibodies, in a significantly larger population of RGCs (Fig. 1I and Fig. 2E,F). In these cells, Brn3c^AP^ staining is apparent in the cell body, but not the dendritic arbor, and they disappear from *Brn3b^−/−^* but not *Brn3b^+/−^* retinas.

**Figure 8:**
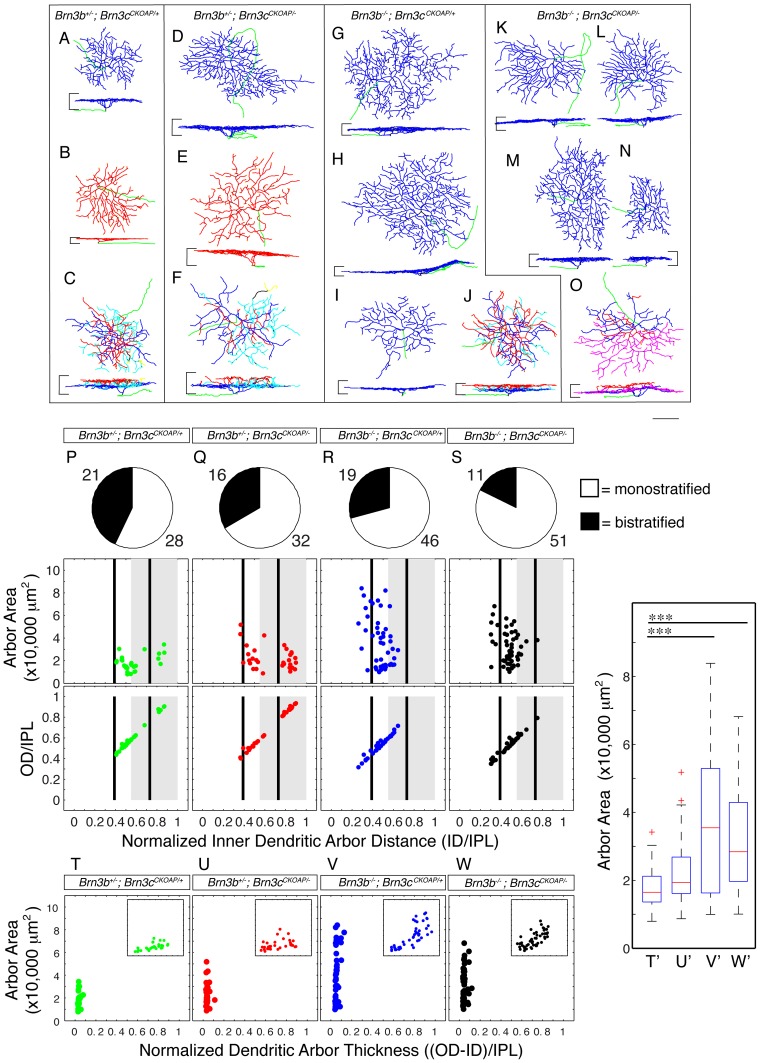
Loss of Brn3b results in specific ablation of “OFF” Brn3c^AP^ RGCs and significant increase in “ON” Brn3c^AP^ RGCs dendritic arbor areas. A–O, Morphological reconstructions of Brn3c^AP^ RGCs from the area of sparse recombination in retinas of genotypes identical to Fig. 2E–H (for numbers of mice for each genotype, see Fig. 2). For each indicated genotype, several examples are shown, surrounded by black outlines. Scale bar is 50 μm. P–W, Quantitations of morphological parameters for Brn3c^AP^ RGCs. The conventions for graphic representations, depicted morphologies and quantitated parameters are as described in Fig. 6. T–W, the extent of lamination depth of dendritic arbors within the IPL is expressed normalized dendritic arbor thickness, and plotted against arbor area. In each panel, the insets show data for excentricity of the cell body (x axis) plotted against dendritic arbor area (y axis), showing significant correlation between the two parameters. Note that “OFF” Brn3c^AP^ RGCs identified as red dendritic arbors in the reconstructions, and data points in between the “OFF” black line and the right (outer) edge of the “OFF” IPL in the scatter plots are completely missing from retinas mutant for Brn3b alone or Brn3b and Brn3c. In retinas from these genotypes, “ON” Brn3c^AP^ RGCs, marked as blue dendritic arbors in the reconstructions, and data points in between the “ON” and “OFF” black lines in the scatter plots, exhibit a marked increase in area. T'–W' represent box-whisker plots for dendritic arbor areas of monostratified RGCs of same genotypes as T–W. RGC dendritic arbor area averages, in μm^2^, are: T' = 17736, U' = 22325, V' = 37548 and W' = 31622 (significance ^***^p<0.001).

In adult retinas, only one dendritic arbor morphology is common to both Brn3b^AP^ and Brn3c^AP^ RGCs, an “OFF” alpha morphology with dendritic arbors stratifying in between the “OFF” Choline Acetyl Transferase (ChAT) band and the INL [47]. This type is completely lost from Brn3c^AP^ RGCs in retinas missing Brn3b (compare reconstructed examples in Fig. 8A–O and quantitations in bottom panels of Fig. 8P–S). In addition, a relative reduction in “ON-OFF” Brn3c^AP^ RGCs (Fig. 8C) and a significant increase in area of “ON” monostratified RGC dendritic arbors (compare Fig. 8G–I and K–N to Fig. 8A,D; areas plotted in Fig. 8P–S middle row and quantitated in Fig. 8T'–U') are observed with decreased dosage of Brn3b and Brn3c.

Thus, Brn3b mutation seems to completely remove the Brn3c^AP^ RGC morphology that normally would express the gene (“cell autonomous effect”), and also affects the morphology of another Brn3c^AP^ RGC, which normally does not express Brn3b in the adult (“non-cell autonomous effect”). Given that the total Brn3c^AP^ RGC numbers are reduced in *Brn3b^−/−^* retinas, dendritic arbor area increase in “ON” Brn3c^AP^ RGCs could be explained by similar arguments as for the Brn3b KO effects on Brn3a^AP^ RGC area changes.

### RGCs surviving Brn3a–Brn3b DKO ablation may be under the transcriptional control of Isl1

**Figure 9:**
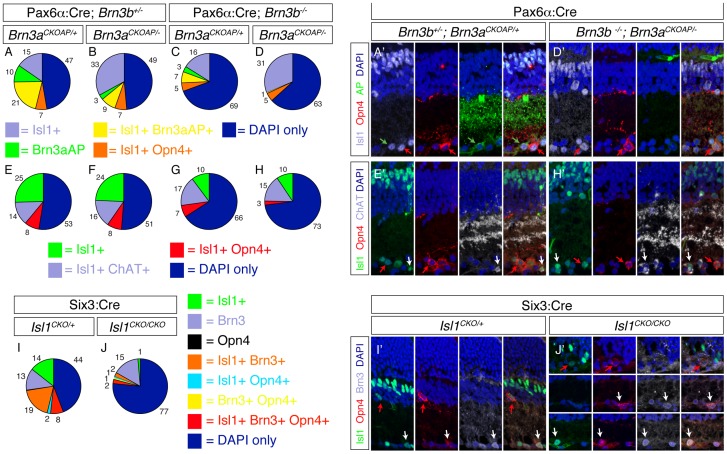
A population of ipRGCs, which survive combined loss of Brn3a and Brn3b, might be transcriptionally regulated by Isl1. A–D', Retinal sections from genotypes identical to Fig. 2 A–D were triple stained for Isl1, Opn4 and Brn3a^AP^. A–D, Pie charts showing relative frequencies of single and double stained cells. No Opn4+ only, Brn3a^AP^+ Opn4+ or triple labeled cells were observed. A',D', Examples of stainings quantitated in A and D show each fluorescent channel merged to DAPI, (panels 1–3) and all channels merged in 4. Note colocalization of Isl1 with either Brn3a^AP^ (green arrow) or Opn4 (red arrow). E–H', Quantitation (E–H) and examples (E', H') of retinal sections triple stained for Isl1, Opn4 and ChAT. Note that Isl1+ (RGCs) and Isl1+ Opn4+ (ipRGCs – red arrows) cells are reduced in numbers in Brn3b KO or Brn3a–Brn3b DKO retinas, but Isl1+ ChAT+ (Starburst amacrines – white arrows) frequencies remain relatively unaffected. I–J', Retinal sections triple stained for Isl1, Opn4 and Brn3. One example from *Six3: Cre; Isl1 ^CKO/+^* control (I') and three examples from *Six3*: Cre; *Isl1 ^CKO/CKO^* (J') mice are shown. Green, red and white arrows point at double labeled cells. I, J pie charts of single, double or triple labeled cells. Note that, in Isl1 KO retinas, total Brn3+ RGCs are reduced to about half and Opn4+ cells are essentially missing. A total of 9 sections for *Six3: Cre; Isl1 ^CKO/+^* control and 11 sections from *Six3*: Cre; *Isl1 ^CKO/CKO^* mice were analyzed. Mean Opn4/DAPI ratios were 10.63% for *Six3: Cre; Isl1 ^CKO/+^* and 0.63% for *Six3*: Cre; *Isl1 ^CKO/CKO^* (t test p value  = 2.4 e-7). In Six3: Cre; *Isl1^CKO/CKO^* retinas rare Isl1+ are observed as result of a low level of mosaicism of the Six3: Cre transgene. Some of these are also Opn4 positive, as seen in panel J', top and bottom row. Scale bar in J' is 25 μm.

Our results show that a small but sizeable number of RGCs survive in Brn3a–Brn3b DKO retinas. At least some of them are NFL and/or Melanopsin positive and their morphologies range from normal with some closely resembling M1 ipRGCs [7], [10], [12] (Fig. 6M, N), to dramatically abnormal ones with large multi-stratified arbors covering the entire depth of the IPL (Fig. 6L). These cells do not express Brn3c since Brn3a–Brn3b DKO retinas are completely Brn3c negative (Fig. 5D). Therefore we asked what transcription factor might be responsible for their survival and/or specification. Recently, genetic interactions were found between the lim homeodomain transcription factor Isl1 and Brn3b during RGC development [28], [29], [60], [67] and Isl1 and Brn3a during DRG and trigeminal development [68], [69]. Also, an immunolocalization survey demonstrated that virtually all ipRGCs express Isl1 [62]. We therefore assessed the status of Isl1 expression in the Brn3a–Brn3b DKO retinas and controls (Fig. 9A–H').

In the GCL of control retinas, Isl1 is expressed in around 60% of Brn3a-AP RGCs, and all Opn4+ ipRGCs (Fig. 9A–D, A', D'). In the Brn3bKO and Brn3a–3b DKO, about 66% of Isl1 positive cells are still left in the GCL, and the few surviving Opn4 positive neurons are Isl1 positive. Triple stains with Isl1, ChAT (a starburst amacrine marker), and Opn4 show that Isl1 only, Isl1 + ChAT + and Isl1 + Opn4 + cells are found in the GCL of *Brn3b^−/−^; Brn3a^CKOAP/−^* retinas, indicating survival of an additional Isl1 + population, distinct from ipRGCs and starburst amacrine cells (Fig. 9E–H, E', H'). It is possible that this population consists of other Brn3-independent RGCs.

Since ipRGCs that survive combined loss of Brn3s are Isl1 positive we asked whether their differentiation is dependent on Isl1, by staining Six3: Cre; *Isl1^CKO/CKO^* retinas, and Six3: Cre; *Isl1^CKO/+^* controls, with Isl1, Brn3, and Opn4 (Fig. 9I, J, I', J'). We find that in Six3: Cre; *Isl1^CKO/+^* retinas Opn4 positive ipRGCs represent around 10% of all GCL cells, and express Isl1 or both Brn3 and Isl1. In contrast, Opn4+ Isl1- RGCs represented only 0.6% of DAPI positive cells in Six3: Cre; *Isl1^CKO/CKO^* GCLs. Interestingly we were able to identify only 15 Opn4+ cells in 8 complete retina sections, three of them being Brn3b +, three Isl1 +, and nine Brn3b + Isl1 +. The presence of Isl1 positive cells in Six3: Cre; *Isl1^CKO/CKO^* is due to a low level of variegation in the Six3: Cre transgene resulting in rare unrecombined, Isl1 + cells. Thus ipRGC development or at least Melanopsin expression seems to largely depend on Isl1.

Since Isl1 has been previously implicated in the development of a large fraction of RGCs, bipolar cells and starburst amacrine cells [28], [29], [67], clearly this transcription factor is not specifically dedicated to ipRGC development. Most likely, it is required in combination with other transcriptional regulators and signaling molecules for the development of these cells.

## Discussion

We find that Brn3 transcription factors interact additively with respect to RGC cell class generation, but have distinct effects on the specification of the morphological features necessary to establish RGC cell types.

Specifically, Brn3b affects Brn3a and Brn3c positive RGCs in cell autonomous and non-cell autonomous fashion. Thus Brn3b loss affects survival, and axon and dendritic arbor features (area and lamination) of Brn3a positive RGCs, including those that normally do not express Brn3b in the adult. Amongst Brn3c^AP^ RGC types, Brn3b results in selective loss of the OFF type, enlargement of dendritic arbors of the ON type, and no visible changes in the ON-OFF type, both of which are Brn3b negative in the adult. In addition, we find that Brn3a loss results in a decrease in overall RGC numbers, thus suggesting that the bias towards bistratified morphologies previously reported is more likely a result of loss of some of the monostratified Brn3aAP RGCs. We also find additive effects of Brn3a – Brn3b but not Brn3b – Brn3c and Brn3c – Brn3a double knock-outs with respect to Brn3^AP^ RGC survival. Amongst the very small fraction of RGCs surviving Brn3a – Brn3b double ablation, we identify ipRGCs which are positive for the transcription factor Isl1. In contrast to their survival in Brn3 null retinas, we find that all ipRGCs are absent from Isl1 KO retinas. These findings support a transcriptional combinatorial code in which the three Brn3 transcription factors, in conjunction with other factors such as Isl1, are responsible for RGC type specification.

### Distinct effects of Brn3 family members on RGC survival and morphology

In the absence of RGC type-specific markers, we have used a genetic marking methodology to describe a combinatorial code of Brn3 expression in RGCs, with Brn3a and Brn3b expressed in a majority of RGC types, and Brn3c only in three RGC morphologies. Additionally, we found that RGCs expressing either Brn3a or Brn3b can survive the ablation of the transcription factor, but exhibit morphological defects in either dendritic arbor or axon formation. These observations raised the question of whether these transcription factors actually play a role in the cell type specification of the 15 cell types they are expressed in. Whereas it was previously established that Brn3b is required for the development of a large fraction of RGCs, it was not clear whether these effects were limited to the cell types expressing the transcription factor in the adult.

We now find that Brn3b loss globally affects survival, axon formation, dendritic arbor area and lamination in Brn3aAP RGCs types, including those that are Brn3b negative in the adult. The effect of Brn3b ablation on the three Brn3c^AP^ RGC types is more selective. Indeed, loss of Brn3b results in a cell autonomous ablation of the “OFF” Brn3c^AP^ RGC population, and a non-cell autonomous dendrite arbor enlargement effect on “ON” Brn3c^AP^ RGCs, with only a modest reduction in numbers of “ON-OFF” Brn3c^AP^ RGCs. This combination of cell autonomous and non cell autonomous effects is paralleled by the defects in Drosophila olfactory neuron specification upon mutation of Acj6, the orthologue of the Brn3s [32]. Thus, in both interactions (Brn3b on Brn3aAP RGC and Brn3b on Brn3cAP RGC), the effect of Brn3b loss extends to more RGC types than predicted by the overlap of expression seen between Brn3b and Brn3a or Brn3b and Brn3c in adult RGCs. It is possible that, during RGC development, Brn3b is expressed in and required for the development of a larger fraction of RGCs than predicted by its adult expression profile. Indeed at various stages of development, RGCs positive for only one of the Brn3s seem to exist, based both on counting cells positive for the three transcription factors and on double stains with antibodies against Brn3a and Brn3b [70], [71]. However, it is also conceivable that loss of Brn3b in one RGC population would result in defective signaling from these cells to other uncommitted precursors, therefore biasing RGC type development even for other cells that never express Brn3b. Since we cannot distinguish the individual Brn3a positive cell types at all stages of development it will be hard to differentiate between the two potential mechanisms at this point. Since individual RGC types cannot be tracked at this point in their early developmental stages, it is also hard to determine whether the loss of RGCs is caused by cell death, lack of specification, or both. Previous studies have yielded conflicting results with respect to increases in apoptotic cell numbers in the GCL of *Brn3b^−/−^* mice [50], [71], perhaps because there is a rather high level of normal cell death throughout RGC development. A deficit in RGCs in the absence of Brn3b is seen from the earliest time points of development, as measured for instance by the number of axons present in the optic nerve, which would argue for a defect in specification, at least of the normal axonal process [49], [50].

The effects of Brn3b loss on dendritic arbor size might be mediated via a contact inhibition dependent mechanism. However, Lin et al., 2004 [26] find that SMI-32 positive and M1 ipRGCs are reduced in numbers in both Math5 and Brn3b KO retinas, but do not show significant increases in dendritic arbor area sizes. This observation, apparently contradictory to the enlarged Brn3a^AP^ RGC dendritic arbors we now find in Brn3b KO retinas might be explained by the fact that no ON Alpha RGCs and only some of M1 ipRGCs are Brn3b positive. In addition, the dendritic arbor morphologies Lin et al label with Thy-1 GFP transgenic alleles are mostly large monostratified RGCs, which we also find in both Brn3b^AP/−^ RGCs [15], [47] and Brn3b^−/−^; Brn3a^AP^ RGCs (this study). Collectively these results might then mean that abolition of lateral contact inhibition may not result in enlarged RGC dendritic arbors in all instances when members of an RGC cell type are removed from their respective mosaic, and therefore this mechanism may not suffice to explain the enlarged dendritic arbor areas we observe. In addition, this mechanism does not address the loss of small area and thick lamination RGCs observed in both Brn3b^−/−^; Brn3a^AP/+^ and Brn3b^AP/−^ RGCs.

### What are the specific roles of each Brn3 in RGC development?

The results we report in this study can be grouped in two broad categories. We report on single and double Brn3 KO effects on overall RGC cell numbers. Thus we find that Brn3a has a survival and/or specification role for particular types of RGCs, and we find that Brn3b and Brn3a KOs have additive effects with regard to overall RGC numbers, and confirm previous reports of similar effects with respect to Brn3b – Brn3c double KO retinas. On the other hand we report that loss of Brn3a and/or Brn3b result in distinct changes in neuronal arbor morphology (see above).

Together with our previous work, these results rule out a simple cascade in which Brn3b acts as a master regulator of all RGCs including those that are Brn3a and Brn3c positive, since plenty of Brn3a^AP^ and Brn3c^AP^ RGCs survive Brn3b deletion, and exhibit a range of morphologica phenotypes, from significantly affected, to normal. Our findings also challenge a model in which individual RGC types require specific Brn3 transcription factors, since the range of ablated or affected RGC types in single or double KO retinas does not overlap with the normal expression domain of either Brn3 transcription factor. Whereas we still do not understand how Brn3s control RGC type specification, the data we present significantly advance the field by excluding some of the potential alternative mechanisms. The working model we currently favor is that RGC type specification is achieved through transcriptional regulation of specific cell type features, whose intersection results in unique definition of RGC types, not unlike the model of the terminal selector cassettes proposed for POU domain homologues in C. elegans.

### Transcriptional regulation of RGC type specification beyond Brn3s

How complete is the transcriptional code of RGC type specification? Brn3a-Brn3b DKO retinas, which are also missing Brn3c, still preserve a small number of RGCs, some of which are positive for the broad RGC markers NFL and Isl1, or/and the more restricted ipRGC marker Melanopsin. We find that a fraction of ipRGCs do not depend on Brn3s for survival (this study). However, a majority of ipRGCs express Isl1 [62] and we now show that most, perhaps all Melanopsin expression depends on Isl1. These results, together with the previously published Isl1 – Brn3b and Isl1 – Brn3a genetic interactions argue that Isl1 is part of the combinatorial transcriptional code of RGC type specification. Since Isl1 loss of function affects development of a larger fraction of RGCs than can be explained by loss of ipRGCs, as well as defects in bipolar and amacrine cell types, it is reasonable to speculate that Isl1 alone cannot account for ipRGC specification, but rather is part of a more complex combinatorial code.

This study completes additional pieces of the puzzle of transcriptional regulation of RGC cell type specification. It establishes that, despite heavy overlap of expression and transcription target site specificity, Brn3s have quite distinct roles during RGC development. These roles can be linked in some cases to cell type specific development but generally tend to influence common RGC type properties like dendritic arbor lamination, or axon formation. In addition, we identify Isl1 as an ipRGCs transcriptional regulator, explaining perhaps why these RGC types are resistant to loss of one or multiple Brn3 factors.

It will be interesting to explore the co-expression and genetic interactions of these factors at various stages of RGC development and their role in adult RGC types. In addition, we need to explore the potential roles of other transcription factors like Eomesodermin, Ebfs and Dlx which have been implicated in RGC development in this combinatorial code. Finally, we need to identify the molecules executing these transcriptional codes, and how they participate in the assembly of RGC diversity.

## Supporting Information

Text S1 This a Matlab m file designed by Friedrich Kretschmer, allowing for the 3D display and conversion of neuronal tracings executed under Neuromantic and saved as.swc files. In addition, projections generated can be exported as vector graphics for publication figures.
(M) Click here for additional data file.
